# Lack of sleep as a contributor to obesity in adolescents: impacts on eating and activity behaviors

**DOI:** 10.1186/s12966-016-0428-0

**Published:** 2016-09-26

**Authors:** Jean-Philippe Chaput, Caroline Dutil

**Affiliations:** Healthy Active Living and Obesity Research Group, Children’s Hospital of Eastern Ontario Research Institute, 401 Smyth Road, Ottawa, ON Canada K1H 8L1

**Keywords:** Sleep deprivation, Adiposity, Body weight, Appetite, Eating behavior, Food intake, Physical activity, Exercise, Sedentary behavior, Screen time, Teenagers

## Abstract

**Background:**

Sleep is an important contributor to physical and mental health; however, chronic sleep deprivation has become common in adolescents, especially on weekdays. Adolescents aged 14–17 years are recommended to sleep between 8 and 10 h per night to maximize overall health and well-being. Although sleep needs may vary between individuals, sleep duration recommendations are important for surveillance and help inform policies, interventions, and the population of healthy sleep behaviors. Long sleepers are very rare among teenagers and sleeping too much is not a problem per se; only insufficient sleep is associated with adverse health outcomes in the pediatric population. Causes of insufficient sleep are numerous and chronic sleep deprivation poses a serious threat to the academic success, health and safety of adolescents. This article focuses on the link between insufficient sleep and obesity in adolescents.

**Discussion:**

This “call to action” article argues that sleep should be taken more seriously by the public health community and by our society in general, i.e., given as much attention and resources as nutrition and physical activity. Not only that having a good night’s sleep is as important as eating a healthy diet and being regularly physically active for overall health, but sleeping habits also impact eating and screen time behaviors and, therefore, can influence body weight control.

**Summary:**

Short sleep duration, poor sleep quality, and late bedtimes are all associated with excess food intake, poor diet quality, and obesity in adolescents. Sleep, sedentary behavior, physical activity and diet all interact and influence each other to ultimately impact health. A holistic approach to health (i.e., the whole day matters) targeting all of these behaviors synergistically is needed to optimize the impact of our interventions. Sleep is not a waste of time and sleep hygiene is an important factor to consider in the prevention and treatment of obesity.

## Background

Sleep is recognized as an important contributing factor to physical and mental health in humans, including adolescents. Healthy sleep requires adequate duration, good quality, appropriate timing and regularity, and the absence of sleep disturbances or disorders [1]. However, lack of sleep represents one of the most common and potentially remediable health risks in adolescents, for whom chronic sleep loss has become the norm [2]. It is well-known that developmental shifts in sleep physiology (adolescents have a phase delay of up to 2 h relative to middle childhood but have to wake up early to go to school) and societal pressures shorten the amount of sleep that adolescents get on school nights [3]. Adolescents have also shown a steeper decline in sleep duration over the past decades compared with younger children or adults [4–7]. Data on 690,747 children and adolescents from 20 countries showed a secular decline of 0.75 min/night/year in sleep duration over the last 100 years, with the greatest rate of decline in sleep occurring for adolescents and on school days [4]. This information is important and can help to inform future interventions aimed at improving sleep habits of adolescents (e.g., delaying school start times).

Insufficient sleep as a possible cause of weight gain and obesity has received considerable attention in the media and scientific literature over the past decade [8, 9]. Interest in this topic area has been fueled by the landmark study of Spiegel et al. [10] published in 2004 showing alterations in appetite hormones after partial sleep restriction in young adults. Mechanistic studies have tried to explain the short sleep-obesity association by demonstrating effects on eating and screen time behaviors that accompany insufficient sleep [11]. More recent studies have tried to intervene on sleep and test if improvements in sleep can positively impact health outcomes [12]. Altogether, the accumulating body of evidence in this research area suggests that sleep should be taken more seriously by the public health community and in our society in general, i.e., given as much attention and resources as nutrition and physical activity.

The focus of this “call to action” article is on insufficient sleep in adolescents and its association with weight gain and obesity. Although the paper is mainly geared towards teenagers, most of the content certainly applies to the general population. The present contribution goes well beyond a synthesis of available studies on the topic with possible mechanisms and includes practical recommendations for health professionals and public health policies. Furthermore, the interconnections among sleep, sedentary behavior, physical activity and diet are discussed. It is hoped that people will fully understand the importance of including “sleep” in the package for good health. It should be noted that such terms as insufficient sleep, inadequate sleep, sleep deprivation, short sleep duration, sleep loss and lack of sleep are used interchangeably in this paper as generic descriptive terms only and do not imply specific amounts but rather “less sleep than needed for optimal health and functioning”.

### Too long or too short: what is the right sleep duration for adolescents?

The National Sleep Foundation in the United States recommends that adolescents aged 14–17 years sleep between 8 and 10 h per night to maximize overall health and well-being [13]. These sleep duration recommendations have recently been supported and adopted in Canada [14]. Population-based estimates indicate that approximately one-fourth to one-third of adolescents in USA and Canada sleep less than the recommended amount [15, 16]. About two-third of them adhere to the sleep duration recommendations, suggesting that long sleepers are rare.

The available evidence that helps inform sleep duration recommendations is weak (i.e., mainly based on cross-sectional studies using self-reported sleep duration), suggesting that expert opinion in this research area is needed until we have better studies. For example, there is a clear need for sleep restriction/extension interventions in adolescents that try to determine upper and lower limits of healthy sleep duration (i.e., dose–response curve). Current sleep recommendations tend to suggest that a generalized optimum exists for the population; however, it is possible that different optimal sleep durations exist for different health outcomes [17]. There is also inter-individual variability in sleep needs (e.g., because of genetic differences or sociocultural contexts) and sleeping longer or shorter than the recommended range may not necessarily mean that it will adversely affect health. However, individuals with sleep durations far outside the recommended range may be engaging in behavioral sleep restriction or may have other health problems. Intentionally restricting sleep duration over a prolonged period of time can certainly compromise overall health [13].

A sleep duration range implies that there is a U-shaped relationship between sleep duration and health outcomes. However, a recent comprehensive systematic review including 141 articles concluded that more sleep is better for children and adolescents, i.e., there is no health risk associated with long sleep [18]. Longer sleep duration was associated with lower adiposity indicators, better emotional regulation, better academic achievement, and better quality of life/well-being in that review [18]. Interestingly, a recent consensus statement from the American Academy of Sleep Medicine and Sleep Research Society recommends that adults should sleep 7 or more hours per night on a regular basis to promote optimal health [19]. This 7 or more hours of sleep threshold is in contrast with the range recommendation issued by the National Sleep Foundation proposing between 7 and 9 h of sleep per night in adults [13]. The main reason for using a threshold value instead of a range is explained by the fact that no one can identify plausible physiological mechanisms by which longer sleep might cause poor health [19]. Furthermore, it is increasingly recognized that the two sides of the U-shaped association between sleep duration and poor health outcomes do not mean the same thing [20]. While short sleep is consistently associated with adverse health outcomes, long sleep is generally associated with other health problems (e.g., psychological distress and chronic pain) that may confound the association (so long sleep is not a problem per se). It would not be surprising if a threshold value (instead of ranges of sleep durations) could also be used for other age groups in the future.

Self-reported sleep duration is generally used in epidemiology because it provides several advantages (e.g., cost saving, less burdensome for participants, etc.) and, therefore, sleep duration recommendations rely heavily on subjective data. Self-reported sleep duration generally overestimates actual sleep duration and is subject to recall and/or social desirability bias [21]. In order to provide better estimates of sleep, actigraphy (called accelerometry in exercise science) is gaining popularity and recognition in epidemiological research [22, 23]. More research is needed to establish if associations of insufficient sleep with health outcomes can be replicated using objective measures. With the increase in use of wearable technology, including commercially available wrist-worn accelerometers, the collection of large-scale objective sleep data is more feasible than ever and can offer a legitimate approach to public health surveillance. Objective monitoring of sleep will offer more precise estimates of health risk and is surely an important direction for research and public health.

In summary, although the ideal amount of sleep may vary from one person to another, sleep duration recommendations are important for surveillance and help inform policies, interventions, and the population of healthy sleep behaviors. Healthy sleep guidelines are thus important from a population health standpoint; however, they need to be adapted on a case-by-case basis.

### Causes of insufficient sleep in adolescents

Although sleep disorders, such as obstructive sleep apnea, have increased in recent years in adolescents, behavioral sleep restriction has become pervasive in modern societies with 24/7 availability of commodities and technologies [1]. Factors explaining sleep loss in adolescents are numerous and can be different between individuals, suggesting that intervention strategies aimed at addressing the causal contributors of behavioral sleep restriction in teenagers should be individualized to maximize success [1]. These factors can include modern technologies associated with excessive light exposure and over-stimulation, consumption of wake-promoting substances such as caffeine (e.g., energy drinks), aspects of the physical environment (e.g., pet in the bedroom, excessive noise, and temperature extremes) that hinder sleep, and the low priority given to sleep by families and society in general [1, 2]. Other contributing and potentially remediable factors to consider include excessive demands on students’ time because of homework, extracurricular activities, after-school employment, social activities, and lack of parental monitoring or rules about bedtimes in the household [24].

Declines in adolescents’ sleep duration have occurred as a result of progressive delays in bedtimes, but unchanged wake time [25, 26]. This has resulted in catch-up sleep on weekends as a means to address the accumulated weekday sleep debt [1–3]. Thus, interventions that can target the sleep hygiene of teenagers on weekdays are highly relevant. As an example, the evidence strongly implicates early school start times as a key modifiable contributor to insufficient sleep in adolescents [27, 28]. A growing body of evidence has shown that delaying school start times is an effective countermeasure to chronic sleep deprivation and has a wide range of potential benefits to students with regard to physical and mental health, safety and academic achievement [27, 28].

An important barrier to healthy sleep hygiene in adolescents is the fact that sleep is generally perceived as a waste of time [1]. Our socio-cultural environment (e.g., long store hours, late-night sports events, energy drinks, screen time and artificial light exposure at night) does not promote healthy sleep habits and people living in a 24/7 society place sleep low on their priority list [1]. Sleep health is often neglected as a crucial component of a healthy lifestyle, and sleep deprivation is not currently considered a public health concern by most education or policy makers [1]. Much of the attention is still devoted to nutrition and physical activity as the two main pillars associated with weight stability and good health. However, it is hoped that more attention will be devoted to sleep in the future. Not only that having a good night’s sleep is as critical as exercising every day and eating well for overall health, but sleeping habits also impact eating and screen time behaviors and, therefore, can influence body weight control [29–31].

### Consequences of insufficient sleep in adolescents

An accumulating body of evidence (including large representative cohorts and experimental studies with ecological validity) indicates that chronic sleep deprivation poses a serious threat to the academic success, health and safety of adolescents [2]. Insufficient sleep is associated with negative outcomes in several areas of health and functioning, including obesity, depression, school performance, quality of life, and risk-taking behaviors [18, 32, 33]. Additionally, insufficient sleep is associated with disturbances in cognitive and psychomotor function including mood, thinking, concentration, memory, learning, vigilance and reaction time [34–36]. Insufficient sleep also incurs financial costs relating to health and other expenditures and non-financial costs relating to loss of quality of life and premature death [37].

### Lack of sleep as a contributor to obesity in adolescents: observational evidence

Two systematic reviews and meta-analyses have tried to quantify the cross-sectional association between sleep duration and obesity in the pediatric population. Cappuccio et al. [38] examined whether short sleep duration (generally defined as < 10 h per night) was associated with obesity in 30,002 children and adolescents from around the world (*N* = 12 studies included). They observed a pooled odds ratio (OR) of 1.89 [95 % confidence interval (CI) 1.43–1.68] for short sleep duration and its association with obesity. Similarly, Chen et al. [39] analyzed 11 studies and reported that children and adolescents with shorter sleep duration had a pooled OR of 1.58 (95 % CI 1.26–1.98) for overweight/obesity, and those with shortest sleep durations had an even greater risk when compared with those having longer sleep durations (OR 1.92; 95 % CI 1.15–3.20). Thus, cross-sectional studies consistently report an association between short sleep duration and a higher risk for excess body weight in the pediatric population, including adolescents.

Systematic reviews and meta-analyses examining the longitudinal associations between sleep duration and subsequent weight gain and obesity have also been published. Ruan et al. [40] reported that compared with children/adolescents having the longest sleep duration (~12.2 h), those with the shortest sleep duration (~10.0 h) had relatively larger annual body mass index gain (pooled β coefficient 0.13; 95 % CI 0.01–0.25 kg/m^2^). They also showed that with every 1 h/day increment in sleep duration, the annual body mass index gain was reduced by 0.05 kg/m^2^ (*β* = −0.05; 95 % CI −0.09, −0.01). Twenty-five longitudinal studies were included in this analysis, including 56,584 children and adolescents with an average 3.4-year follow-up period [40]. Fatima et al. [41] also explored the prospective association between short sleep duration and overweight/obesity in 24,821 children and adolescents (*N* = 11 longitudinal studies in their meta-analysis). They observed that participants sleeping for short duration had twice the risk of developing overweight/obesity compared with those sleeping for long duration (OR 2.15; 95 % CI 1.64–2.81). These findings are also in line with the systematic review published by Magee and Hale [42] in 2012 showing a positive relationship between short sleep duration and subsequent weight gain in children and adolescents (*N* = 7 longitudinal studies).

Collectively, there is consistent evidence from observational studies (longitudinal and cross-sectional studies) that short sleep duration is associated with weight gain and obesity in adolescents. However, common limitations generally found in epidemiological studies preclude causal inference between insufficient sleep and weight gain. Important limitations of observational studies in this topic area include: lack of control for important confounders (e.g., depression, psychosocial problems, chronic illness, medications), the reliance on self-reported sleep, and the possibility of bidirectional effects (insufficient sleep causing weight gain and excess body weight causing insufficient sleep), thereby creating a setting for a vicious cycle [43]. Therefore, experimental studies are important and instrumental in demonstrating whether sleep restriction causes weight gain in humans.

### Lack of sleep as a contributor to obesity in adolescents: experimental evidence

Sleep restriction interventions are critical in determining if insufficient sleep can lead to weight gain over time. Most of these sleep experiments have been conducted using short-term follow-up periods and have tried to investigate the mechanisms by which sleep restriction could potentially lead to weight gain [44]. Long-term randomized controlled trials of sleep restriction on the development of obesity are unlikely to happen (for ethical and logistic reasons) and, therefore, short-term sleep restriction experiments are the study design of choice to answer this important question.

Most of the evidence base in this area, i.e., experimental studies showing that sleep restriction leads to weight gain in humans, comes from studies conducted in adult participants. For example, Spaeth et al. [45] reported that sleep-restricted participants tested under controlled laboratory conditions (*N* = 225 healthy adults) gained 1 kg more than controls after 5 consecutive nights of 4 h in bed per night. Sleep-restricted participants consumed extra calories (130 % of daily caloric requirements) and the increased daily energy intake was due to more meals eaten and the consumption of about 550 additional calories during late-night hours. These findings are very similar to another sleep restriction experiment by Markwald et al. [46] showing that 5 nights of 5 h in bed per night led to a 0.82 kg weight gain in 16 healthy adults tested in the laboratory. Here again, energy intake (especially at night after dinner) was in excess of energy needed to maintain energy balance. Interestingly, the authors also observed that transitioning from an insufficient to adequate/recovery sleep schedule decreased their energy intake and resulted in weight loss. In the pediatric population, Hart et al. [47] conducted a randomized crossover trial in 37 children aged 8–11 years and showed that compared to decreasing sleep duration by 1.5 h/night, increasing sleep duration by 1.5 h/night over a week resulted in lower food intake and lower body weight. These findings are encouraging and demonstrate that intervening on sleep duration in the pediatric population is possible and can lead to improvements in appetite control and body weight regulation. However, much needs to be done on how best to improve sleep habits of teenagers. It is also still unknown whether the short-term effects can persist on a chronic basis.

### Mechanisms by which a lack of sleep may lead to weight gain

There is no doubt that the seminal paper published by Spiegel et al. [10] in 2004 generated a lot of interest in this topic area and proposed a hormonal explanation to the short sleep-obesity association. Although they reported significant alterations in key appetite hormones after sleep restriction in adults (e.g., increase in ghrelin, decrease in leptin, and increased sensations of hunger), many subsequent studies that used an ad libitum food access protocol, similar to what can be found under free-living conditions, failed to replicate these findings [11]. It is clear that insufficient sleep is a stressor to our metabolism and induces wide-ranging adverse effects on a variety of body systems and hormones [48]. However, in light of more recent investigations in the field, it is adequate to say that the hormonal explanation is probably not the most important mechanism to explain the association between insufficient sleep and increased food intake [49].

Insufficient sleep has to impact energy intake and/or energy expenditure in order to change body weight. Increased food intake as a result of insufficient sleep is definitively the main explanation to the short sleep-obesity association [11]. In fact, sleep restriction interventions generally report an increase in 24-h energy expenditure of ~5 %, mainly due to the energy cost of wakefulness [46, 50]. However, some individuals may reduce their spontaneous physical activity or even increase their sedentary behavior after sleep deprivation due to fatigue and tiredness [51]. Large inter-individual variations in physical activity have been observed after sleep restriction (some people move less, some move more, and some don’t change anything) [51]. In contrast, there is consistent and robust evidence showing that insufficient sleep increases food intake [52–54], emphasizing that “energy in” is the key mediator of the short sleep-obesity association.

Short sleep duration, poor sleep quality, and late bedtimes are all associated with excess food intake, poor diet quality, and obesity in the modern obesogenic environment [11, 29, 55]. Increased food intake resulting from insufficient sleep seems to be preferentially driven by hedonic rather than hormonal factors [11, 29, 49]. Short sleepers have more time and more opportunities for eating because they spend more time awake. This is consistent with studies showing that short sleepers snack more and consume more meals per day [11, 29]. In addition, short sleepers tend to crave more for energy-dense foods (i.e., high in fat and sugar). For example, studies have shown that sweets and desserts are more appealing to adolescents after sleep restriction [56–58]. These findings are consistent with neuroimaging experiments showing that sleep restriction activates brain regions sensitive to hedonic food stimuli [59–61]. Thus, eating (especially energy-dense foods) may be seen as a normal physiological response to provide energy needed for the brain to sustain additional wakefulness. Given that short sleepers may find it more difficult to resist energy-rich food items in today’s environment, strategies should be proposed to them in order to limit their caloric consumption and risk for eventual weight gain (e.g., healthy snacks).

### Interconnections among sleep, sedentary behavior, physical activity and diet

Research examining the effects of sleep, sedentary behavior, physical activity and eating behavior on health outcomes has mainly been conducted in silos, i.e., independently or in isolation of the other components. This analytical approach is certainly not representative of what happens in real life because health is dependent on the composition of our behaviors [14, 30]. Recent research shows that combinations or patterns of behaviors can impact health in ways that cannot be explained by the effect of individual behaviors alone [62–65]. Not only that these behaviors interact and influence one another but they all matter for optimal health. Messages suggesting that physical activity can offset the adverse effects of a bad diet and/or lack of sleep are ill-founded and certainly not in line with emerging research indicating that all of the above behaviors matter for optimal health. Recent analytical approaches (e.g., compositional data analysis) are instrumental in providing a good understanding of this issue and for determining the best “cocktail” of behaviors associated with improved health.

It is increasingly recognized that a more inclusive and integrated approach is needed to better address current health challenges. Different combinations of these behaviors exist in our society and the best scenario, according to the available evidence, is the following for adolescents: sleeping 8–10 h/night, no more than 2 h of recreational screen time per day, ≥ 60 min of moderate-to-vigorous physical activity per day, and following dietary recommendations [14, 62–65]. This list is certainly not exhaustive but is in line with current guidelines and suggests that the more “healthy behaviors” people can integrate and tolerate in their day the better it is for their health – and they all matter. This holistic approach to health (i.e., the whole day matters) is certainly a shift in our approaches to health that have traditionally been dealt with separate and distinct guidelines [14].

Beyond finding an optimal balance of healthy behaviors over 24 h, they all interact and influence each other. For example, aerobic physical activity has been shown to improve sleep quality [66] and can influence eating behaviors [67]. Screen time has been shown to disrupt sleep [68] and is associated with overconsumption of food [69]. Insufficient sleep can reduce physical activity level due to feelings of fatigue [51], is associated with more screen time late at night [68], and increases food intake [29]. Finally, certain foods may promote sleep or improve sleep quality [70], and the fuel we decide to ingest can certainly influence our performance in sport [71]. Thus, it becomes clearer that public health interventions, guidelines, and accompanying messages should target all of these behaviors synergistically to optimize health.

### Influence of sleep in the treatment of obesity in adolescents

Although maintaining good sleeping habits is increasingly recognized as a good strategy to prevent future weight gain, there is also a growing body of evidence showing that adequate sleep is important to improve the success of weight-loss interventions [31]. Energy restriction combined with increased physical activity is typically used to induce weight loss and improve health markers. However, recent studies indicate that for the same weight-loss program, short sleepers can expect to lose less body fat compared to those who sleep the recommended amount per night [72–74]. For example, a study conducted in obese adolescents who participated in a summer camp-based immersion treatment program showed that incorporating sleep components into weight loss interventions may boost their influence on sleep and ultimately improve the effectiveness of treatment outcomes [75].

The fact that lack of sufficient sleep may compromise the efficacy of common weight-loss interventions is increasingly recognized and accepted by health care professionals and has found its way into clinical practice. For example, the Canadian Obesity Network (the largest obesity organization in the world) launched a set of practitioner tools – the 5As of Obesity Management (ask, assess, advise, agree and assist) – that highlight the importance of addressing sleep for successful weight management [76].

Successful weight management is not an easy task, and a good understanding of the root causes of weight gain and barriers to weight management is crucial to success. Although there is no simple solution, many health professionals don’t even ask questions about sleep [29]; unfortunately, it can be an important confounding factor on the road to success. Sleep should definitively be included as part of the lifestyle package that traditionally has focused on diet and physical activity.

### Sleep interventions and their impact on obesity management in adolescents

A systematic review and meta-analysis of interventions targeting sleep and their impact on child body weight, diet and physical activity has recently been published [12]. Of the eight randomized controlled trials included, three enforced a sleep protocol and five targeted sleep as part of multicomponent behavioral interventions either exclusively or together with nutrition and physical activity. Overall, findings from the included studies suggest that where improvements in child sleep duration were achieved, a positive impact on body mass index, nutrition and physical activity were also observed. This is in line with a recent review of randomized controlled trials which included studies mainly conducted in adults showing that improvements in sleep duration resulted in a significant reduction of 0.54 kg (95 % CI −1.01 to −0.07) in body weight compared to those in the control group [77]. Preliminary observations from a prospective randomized controlled trial to measure the effects of extending sleep on body weight in adult short sleepers with obesity also revealed that participants in the intervention group reported more willingness to exercise and fewer cravings for sweet and salty foods during the evening compared to participants in the control group [78].

Under real-life conditions and without any intervention targeting sleep, findings from our group showed that a spontaneous change in sleep duration from a short to a healthier amount of time was associated with attenuated gain in fat mass over time compared to those who maintained their short sleep duration habits [79]. However, increasing sleep duration may not be feasible for some people for various reasons. Trying to address the barriers of insufficient sleep at the individual level is important to maximize success. As discussed earlier in this manuscript, declines in adolescents’ sleep duration have occurred as a result of progressive delays in bedtimes on weekdays, but unchanged wake time [25, 26]. Recent results from a representative sample of adolescents showed that later bedtimes during the week from adolescence to adulthood was associated with an increase in body mass index, highlighting adolescent bedtimes as a potential target for weight management [80]. From a public health perspective, delaying school start times would certainly be an important countermeasure to insufficient sleep in teenagers [27, 28]. At the individual level, removing electronic screen devices from the bedrooms has been shown to be an effective strategy to improve sleep [81]. Many adolescents engage in screen time within the hour before bedtime or even use their cell phone in bed, which delays sleep onset and reduces their sleep quality [82]. Many teenagers are addicted to their cell phone and solutions to reduce screen time before bedtime are not easy to implement in today’s environment. At the very least, self-luminous display screens that decrease circadian stimulation are available (or cell phone applications that change the blue light coming from the screen to an orange light towards the end of the day).

### Why public health efforts should better promote the benefits of a good night’s sleep for overall health?

From a public health perspective, there is minimal risk in taking a pragmatic approach and promoting a good night’s sleep for overall health. Recent research has demonstrated that sleep is not only a major determinant of health but also interacts with eating and activity behaviors to ultimately impact health [11, 29]. Neglecting sleep in the health assessment can certainly undermine the success of our interventions to improve overall health, especially in individuals having poor sleeping habits.

As recently discussed, few health professionals have the opportunity to learn about sleep and its impact on health [83]. Including sleep questions in health assessments and lifestyle modification interventions does not need to be complicated and only very simple questions can provide a clear picture of whether the individual has good or poor sleeping habits in general. This is especially important for dietitians and kinesiologists, because insufficient sleep has been shown to increase food intake and is associated with more screen time, thereby rendering adherence and compliance to behavior changes more difficult. Family doctors and other health care professionals dealing with behavior modification should also assess sleep hygiene.

Table 1 provides a sample of questions that can easily be incorporated into any health and lifestyle assessment. The three key sleep dimensions that need to be assessed are: sleep duration, sleep quality, and sleep timing. All of these sleep dimensions can impact health indicators independently of each other, suggesting that all three need to be met in order to say that someone has good sleep. Then, finding and trying to address the root causes and barriers of a possible inadequate sleep pattern need to be done. Finally, health professionals should determine when referral to a sleep specialist is required. Patients describing symptoms that align with a sleep disorder (e.g., obstructive sleep apnea) should be referred to a sleep specialist.

**Table 1:** Examples of sleep questions that can be included in the health and lifestyle assessment

Questions	Desired answers
1. What time do you go to bed every night and wake up every morning?	Consistent (even on weekends)
2. How many hours do you sleep on an average night?	8–10 h (adolescents)
3. Do you have difficulty falling asleep once in bed?	No, usually I fall asleep within 30 min
4. How many times do you wake up each night?	Never or once per night
5. Do you feel refreshed upon waking in the morning?	Yes
6. How often do you feel sleepy during the day?	Never or rarely

Adapted from Golem et al. [83]

### Future research priorities

Below is a list of five research priorities that need to be achieved to move forward with this field of research.

- Replicate findings observed with self-reported sleep with the use of objective measures (e.g., actigraphy/accelerometry)
- Examine the effects of screen exposure before bedtime on sleep, eating behavior and body weight
- Conduct more sleep restriction/extension studies, with various outcome measures, to have a better sense of dose–response curves and thereby better inform sleep duration recommendations
- Determine if the adverse effects of insufficient sleep on health can be offset or attenuated by other behaviors (e.g., physical activity)
- Assess the feasibility of increasing/improving sleep in teenagers and identify key elements of success.

## Conclusions

Sleep is an essential component of healthy development and is required for physical and mental health. However, declines in adolescents’ sleep duration observed in recent decades are concerning and it is time that we make better sleep a national health priority. In our busy, work-obsessed society, sleep is seen as a luxury or a waste of time. There is strong evidence demonstrating that insufficient sleep leads to obesity and is associated with a long list of adverse health problems. Lack of sleep increases food intake and is associated with more screen time. A good night’s sleep can improve the success of weight loss interventions by facilitating appetite control and increasing physical activity level in some individuals. Health care providers should include sleep questions in their health assessment, especially if the behavior modifications target diet and screen time. Overall, it is hoped that the lack of awareness among the public and some health care professionals about the health significance of sleep will change in the future – Let’s take sleep more seriously!

## Abbreviations

OR: Odds ratio
CI: Confidence interval
